# Exploring fellowship programs: Key information for nurses

**DOI:** 10.1097/NSG.0000000000000224

**Published:** 2025-06-23

**Authors:** Tracy Jones-Darnell

**Affiliations:** **Tracy Jones-Darnell** is a Professor at Nightingale College in Salt Lake City, Utah.

## Abstract

Fellowship programs can help nurses who want a change but remain dedicated to the nursing profession. This article discusses the different types of fellowships, typical requirements, and recommendations for nurse leaders to support nurse fellowship programs.

**Figure:**
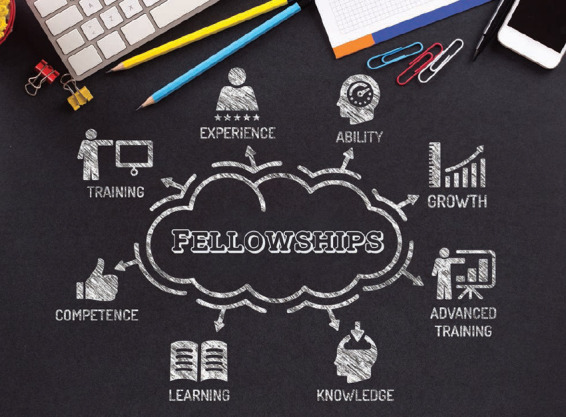
No caption available.

Since the COVID-19 pandemic, the number of nurses leaving the profession has increased nationwide.^1^ This increase can be attributed to retirement, with the average age of a nurse being 52, although moral distress and burnout have also commonly been reported.^2^ If asked, most nurses will likely say that nursing is their calling. As such, leaving the profession is not an easy decision, especially since they have invested years into training, education, and passing the standardized NCLEX. In lieu of leaving the profession altogether, nurses could instead consider going into a different specialty or practice area. Fellowship programs can assist nurses who want a change but are still dedicated to the nursing profession.

Nurse fellowship programs may be the solution for nurses who are burned out in their current position on their unit. A nursing fellowship is typically a paid position that nurses must apply for as they would a new job. These programs are designed to transition experienced nurses who want to advance their skills in a new specialty or practice area.^3^ For example, if an experienced nurse wants to change specialties from the OR to women's health, or a nurse who has worked in the ED decides to enter psychiatry to have a slower-paced work life, they would need training and orientation in the new practice area and specialty. Nurse fellowship programs can last up to 1 year and consist of classroom education followed by supervised clinical training. Fellowship programs are offered in many states and healthcare facilities across the country.

Nursing students are afforded experiences with each specialty during nursing school. However, whereas some of these experiences last a semester, others may be a day or are just a few weeks without clinical experience. The historic Future of Nursing (FON) report recommended advanced training for nurses and highlighted the link between quality of care and advanced training programs.^4^ Fellowships enable nurses to learn critical nursing competencies and expand their clinical judgment. This new knowledge not only improves the quality of care for patients but also increases the nurses' value to the employer due to expertise in another specialty. Nurses can build personal and professional networks to enable collaboration between specialties.

**Table:** Examples of nursing fellowship programs

Program and location	Degree to apply	Requirements	Experience	Program length	Website
UT Southwestern Memorial Center, Dallas, TX	BSN	- TX RN license - BLS - Résumé	6 months of nursing experience	6 months	https://jobs.utsouthwestern.edu/nursing-residency/#tab-id-2
Children's Health, Dallas, TX	BSN	- TX RN license - BLS - Résumé	1 year of clinical experience	6 months	www.childrens.com/for-healthcare-professionals/education-training/nurse-residency/rn-fellowship
Mayo Clinic School of Health Sciences, Perioperative Nursing, Arizona	BSN preferred	- Arizona RN license - BLS - CV/résumé - Interview	1 year of clinical medical or surgical experience	- 40 contact hours - 16 college credits - can sit for Certified Perioperative Nurse (CNOR) examination after 2 years	https://college.mayo.edu/academics/health-sciences-education/fellowship-in-perioperative-nursing-arizona/application-process/
Carilion Clinic Wilderness Fellowship	NP	- Board certified NP - 2 letters of recommendation - CV - Personal statement	2 years of postgraduate clinical experience	- 12 months	www.carilionclinic.org/gme/wilderness-medicine-np-pa-fellowship#about

## Types of fellowships

There are numerous opportunities for fellowship programs, and they provide nurses with mentoring, education sessions, and hands-on clinical experiences (see *Examples of nursing fellowship programs*). Most US-based fellowship programs are located in and facilitated by hospital systems.

Nursing fellowship programs cover a wide variety of focus areas and specialties. There are nursing fellowship programs that focus on developing nurse executives and senior nurse leaders^5^ as well as perioperative fellowship programs for nurses interested in transferring from another specialty.^6^ There are also programs available in long-term care (LTC), long-term acute care, and rehabilitation programs, although these are fewer. There are also policy fellowships that introduce frontline nurses to understanding how to influence health policy.

For frontline nurses' transition, there are fellowship programs that enable experienced medical/surgical nurses to transition to becoming an OR nurse or a neonatal ICU (NICU) nurse. Some fellowships focus on experienced nurses who want to transition from a nonacute care practice area, such as LTC, behavioral health, management, primary care, or prison health, to the ED. In addition, some Magnet^®^ hospitals offer research fellowships that teach frontline nurses how to ask research questions, review the literature, and participate in a research team with a nurse scientist.

Some fellowship programs are designed particularly for nurses with graduate degrees. Graduate fellowships are traditionally programs where a nurse with a graduate degree can transition to an area of practice that may be vastly different. For example, an NP may want to transfer from a medical/surgical specialty to the OR or NICU; there are also fellowship programs for NPs in Wilderness Medicine.^7^ Nurses with a graduate degree in administration may want to complete a government affairs fellowship to learn more about the political process and nurse advocacy. A nurse with a graduate degree in leadership will complete a 1-year administrative fellowship to gain experience. A new APRN will complete a fellowship program to gain experience in the role while having a mentor and a more structured, supportive transition process for entry into practice.

Fellowship programs can last anywhere from a few months up to a year and are generally a combination of both clinical and classroom experience. Some fellowship positions are paid, while others are not. Of note, fellowship programs are less common than internships or nurse residencies for new graduate nurses.

Although there is no generalized website or location to read about all the nursing fellowship programs in the US, there are multiple resources to explore, such as professional nursing organizations, governmental agencies, and healthcare systems within the state.

## Typical requirements

Program requirements vary depending on the state and facility. To be accepted into a fellowship program, nurses apply just as one applies for a job. To be considered for a fellowship program, one must be an RN who graduated from an accredited nursing program and possesses a valid nursing license. Other requirements may include a minimum GPA from nursing school transcripts, a curriculum vitae or résumé, a cover letter, letters of recommendation, an essay, and background checks.

## Recommendations for nurse leaders

Nurse leaders can advocate for the healthcare system to start a fellowship program for seasoned nurses. Nurse fellowship programs must be committed to education and diversity to develop nurses who represent our diverse community and patient population. The mentoring in fellowship programs is geared toward providing a customized learning experience and offers an opportunity for program fellows to work closely with experts and connect with other nurses. This support system of subject matter experts can help program fellows throughout the learning process and career transition. Other essential values of fellowship programs include building and developing leadership and innovation skills to advance the nursing profession.

As nurses experience different milestones in life, their professional needs may also change. Nurse leaders need to create opportunities for nurses to remain in the profession and be able to transition into roles that are more appealing or safer for their station in life. Nurses need strategies that will enable change as their interests, motivations, and physical capabilities evolve over time. Every nurse should be in control of the path forward in their nursing career trajectory. With nursing fellowships, nurses and graduate nurses can deepen their clinical competencies, broaden their knowledge in specialty areas, and pursue their passion.
